# Supplementary material for: Host shifts result in parallel genetic changes when viruses evolve in closely related species
Source: PLoS Pathog. 2018 Apr 12;14(4):e1006951. doi: 10.1371/journal.ppat.1006951 (PMC5897010; doi:10.1371/journal.ppat.1006951)
Supplement: S1 Supporting information — (PDF) [file ppat.1006951.s001.pdf]

Supplementary methods

Species	Food
D.americana	malt
D.arizonae	banana
D.euronotus	cornmeal
D.lummei	malt + yeast
D.mauritiana	proprionic
D.melanogaster	cornmeal
D.mojavensis	banana
D.montana	malt + yeast
D.paramelanica	cornmeal
D.persimilis	malt
D.prosaltans	proprionic
D.pseudoobscura	banana
S.lebanonensis	proprionic
S.pattersoni	Banana
D.santomea	cornmeal
D.sechellia	proprionic
D.simulans	cornmeal
D.teissieri	cornmeal

Table S1: Fly species and food reared on. Food recipes can be found in [1, 2]

Primer pair code	Primer	Sequence	Annealing temperature °C
A	DCV62F	GCCTGCGGTCCCTAATTGTTGAAT	69
	DCV1245R	CATTCATCTGTTCAGAGCCAACACCAT	
B	DCV1131F	GAGTTGCGTCGACAAATCAAAAATAGGAA	69
	DCV2330R	CTGAGGAAAAGTGTTGCAAGAGCGAAT	
C	DCV2243F	TGATGATGCGTTTCAGAAGAAAGACGA	69
	DCV3406R	GTAGACCACTGGGCAAGTTCACGTTTC	
D	DCV3290F	CGAATGTGGTATGCATAGGATGTCTGC	69
	DCV4468R	ACTGCCTGTCCCACACGAATAGATGAC	
E	DCV4377F	CCCAATATGGTTGATCCACTTGGTGAT	69
	DCV5495R	AAATTTCTCCATCACACGAATCCACGA	
F	DCV5405F	ACCTTCCGGCAACCCTTTCACTGTTAT	69
	DCV6553R	GACTAAAGTTGCTCGCAAACCCACAAA	
G	DCV6423F	CTGGTCTTTGGTCTTCCGCTACAACTG	69
	DCV7693R	GCGAGAAACATCGGGAACTCCTGTAG	
H	DCV7449F	TACTTGGAACACAACCGATGCCACTAG	71
	DCV8902R	AGGATCCAGAATAGTAAGGGAGTTAGGGT	
I	DCV8153F	CATGGTGTTCATCCAATGACCATAGACAC	70
	DCV9039R	CCGTGTAAGCAGGGCAGATAGTTACTGAA	

Table S2: Primers used to amplify DCV genome. PCR Cycle: 98°C 30sec, (98°C 30sec, 69-71°C 30sec, 72°C 45sec) x 35 cycles, 72°C 2min

Supplementary results

Distribution and types of mutations

DCV contains two open reading frames (ORF) that cover 88% of our reference DCV genome (Figure 1). ORF1 codes for non-structural proteins and is cleaved into eight putative proteins, including the RNA dependant RNA polymerase, a protease-like protein (3C), helicase proteins, as well as DCV-1A that acts as a suppressor of antiviral RNAi [3-8]. ORF2 codes for the four structural proteins that form the capsid. We examined whether SNPs that arose in parallel were more likely to be in coding regions and whether these SNPs were synonymous or non-synonymous.

ORF1 contained 391 SNPs (0.074 per site) and ORF2 contained 217 SNPs (0.080 per site). Of the 56 polymorphic sites with a significant signal of parallel evolution within the same host species ($P<0.05$; significantly parallel SNPs at frequencies >0.05 in two lineages are shown with a red asterisk in Figure 1), 91% were within the open reading frames; 29 in ORF1 (0.005 per site), and 22 in ORF2 (0.008 per site). The significantly parallel SNPs were not significantly more likely to be in an ORF was than the non-significant SNPs (51 of 56 vs 477 of 586, Chi-sq = 0.203, df = 1, p-value = 0.65).

We found 52% of significantly parallel SNPs (counting both alleles separately at tri-allelic sites) in ORF1 were non-synonymous (17/33) compared to 51% of non-significant SNPs (181/358). In ORF2 we found 59% of significantly parallel SNPs were non-synonymous (16/27) compared to 50% non-significant SNPs (95/190). There was no difference in the proportion of non-synonymous SNPs that were significantly parallel compared to those non-significant SNPs (ORF1: Chi-sq = 2.18, df = 1, P = 0.14; ORF2: Chi-sq = 0.47, df = 1, P= 0.49; both ORFs combined: Chi-sq = 3.06, df = 1, P= 0.08). However, we note that even synonymous mutations may alter fitness in an RNA virus [9].

Supplementary figures

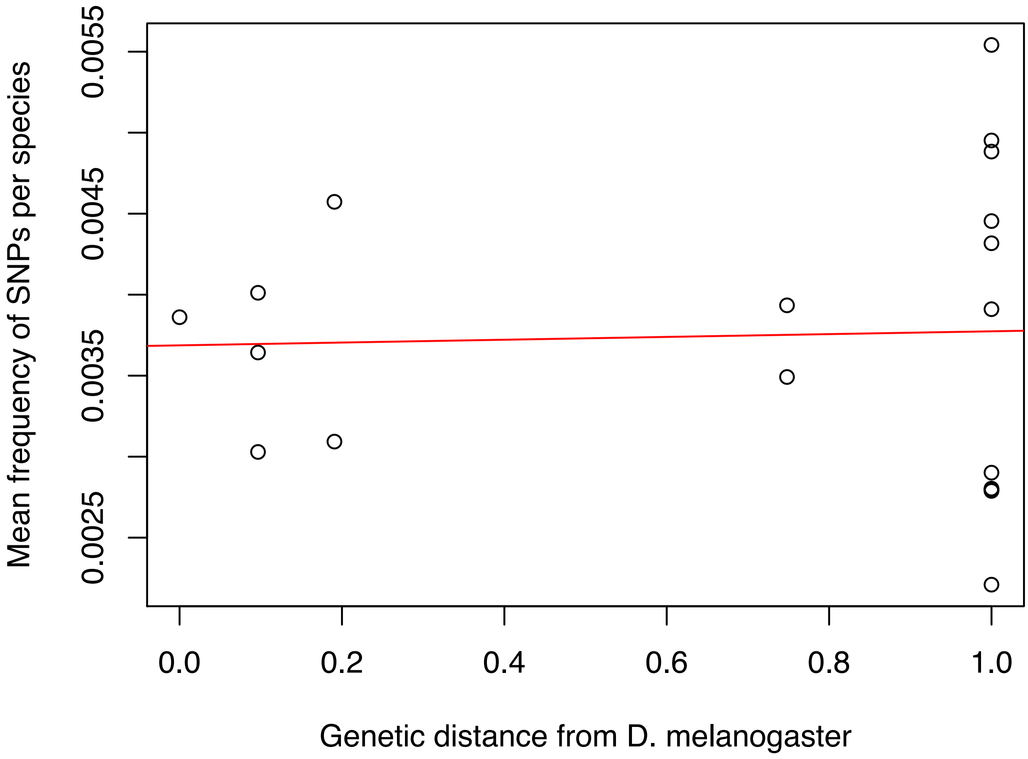

Figure S1: Correlation between mean SNP frequency and distance from *D. melanogaster.* We calculated the mean frequency of SNPs across the viral genome for each host species and examined if viruses showed higher rates of molecular evolution with increasing distance from *D. melanogaster*; the host DCV was isolated from and naturally infects. We found no significant change in mean SNP frequency with distance from *D. melanogaster* (phylogenetic mixed model implemented in MCMCglmm package in R as in [1], correlation= 0.0006 (CIs= -0.0176, 0.0188).

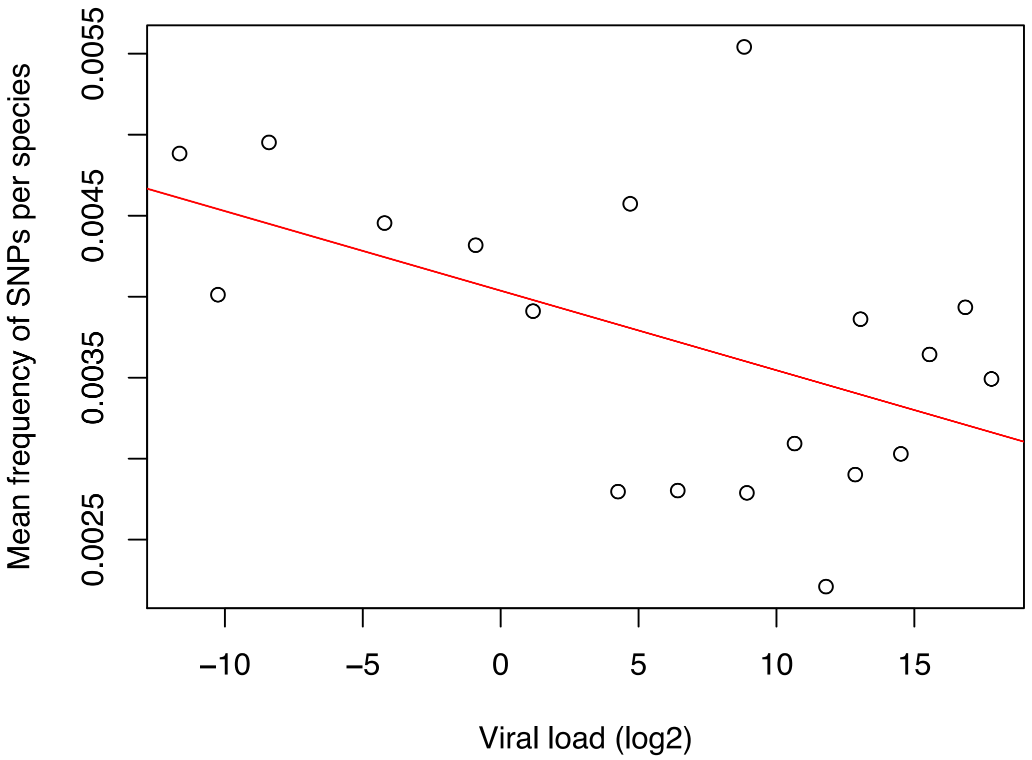

Figure S2: Correlation between mean SNP frequency and RNA viral load.
We examined if mean SNP frequency in a species was affected by viral population size. We used RNA viral load calculated by qRT-PCR as in [1] at passage 10 as a proxy for viral population size (viral load did not change significantly between passages 3 and 10). After correcting for phylogenetic effects of the host species, we found no significant relationship between mean SNP frequency and viral load (phylogenetic mixed model implemented in MCMCglmm package in R as in [1], correlation= -0.00006 (CIs= -0.00072, 0.00054).

Supplementary references

1. Longdon B, Hadfield JD, Day JP, Smith SC, McGonigle JE, Cogni R, et al. The Causes and Consequences of Changes in Virulence following Pathogen Host Shifts. PLoS Pathog. 2015;11(3):e1004728. Epub 2015/03/17. doi: 10.1371/journal.ppat.1004728. PubMed PMID: 25774803.
2. Longdon B, Hadfield JD, Webster CL, Obbard DJ, Jiggins FM. Host phylogeny determines viral persistence and replication in novel hosts. PLoS Pathogens. 2011;7((9)):e1002260. doi: 10.1371/journal.ppat.1002260.
3. Jan E. Divergent IRES elements in invertebrates. Virus Res. 2006;119(1):16-28. doi: 10.1016/j.virusres.2005.10.011. PubMed PMID: 16307820.
4. Johnson KN, Christian PD. The novel genome organization of the insect picorna-like virus Drosophila C virus suggests this virus belongs to a previously undescribed virus family. J Gen Virol. 1998;79 (Pt 1):191-203. Epub 1998/02/14. PubMed PMID: 9460942.
5. Nakashima N, Nakamura Y. Cleavage sites of the "P3 region" in the nonstructural polyprotein precursor of a dicistrovirus. Arch Virol. 2008;153(10):1955-60. doi: 10.1007/s00705-008-0208-5. PubMed PMID: 18810573.
6. Nakashima N, Uchiumi T. Functional analysis of structural motifs in dicistroviruses. Virus Res. 2009;139(2):137-47. doi: 10.1016/j.virusres.2008.06.006. PubMed PMID: 18621089.
7. van Rij RP, Saleh MC, Berry B, Foo C, Houk A, Antoniewski C, et al. The RNA silencing endonuclease Argonaute 2 mediates specific antiviral immunity in Drosophila melanogaster. Genes & development. 2006;20(21):2985-95. PubMed PMID: ISI:000241767900009.
8. UniProtKB [cited 2017]. Available from: http://www.uniprot.org/uniprot/O36966.
9. Cuevas JM, Domingo-Calap P, Sanjuan R. The fitness effects of synonymous mutations in DNA and RNA viruses. Mol Biol Evol. 2012;29(1):17-20. doi: 10.1093/molbev/msr179. PubMed PMID: 21771719.
